# Relative contribution of neuromuscular activation, muscle size, and muscle quality to maximum strength output of the thigh muscles in young individuals

**DOI:** 10.14814/phy2.15563

**Published:** 2023-01-03

**Authors:** Akito Yoshiko, Kohei Watanabe, Hiroshi Akima

**Affiliations:** ^1^ Faculty of Liberal Arts and Sciences Chukyo University Toyota Japan; ^2^ School of Health and Sport Sciences Chukyo University Toyota Japan; ^3^ Research Center of Health, Physical Fitness & Sports Nagoya University Nagoya Japan; ^4^ Graduate School of Education and Human Development Nagoya University Nagoya Japan

**Keywords:** muscle size, muscle strength, neuromuscular function, non‐contractile tissue

## Abstract

This study aimed to investigate the relationship between maximal muscle strength and neuromuscular activation, muscle size, and quality of quadriceps (QF) and hamstring muscles (HM). The study included 24 young men and women. The neuromuscular activation parameter was recorded using a single‐channel surface electromyography (EMG) with the root mean square (RMS) during maximal isometric knee extension and flexion from four muscles: rectus femoris and vastus lateralis for QF; biceps femoris and semitendinosus for HM. In addition, the peak torque was measured during the same session. B‐mode ultrasonographic transverse images were obtained from the anterior, lateral, and posterior thighs. Furthermore, we calculated the muscle thickness (MT) and echo intensity (EI) of the four muscles as indicators of muscle size and quality. The averaged MT, EI, and absolute RMS of QF were calculated by averaging the values of the rectus femoris and vastus lateralis, and that of HM was calculated by averaging the values of the biceps femoris and semitendinosus. The knee extension peak torque was correlated with EI (*r* = −0.61, *P* < 0.01) and RMS (*r* = 0.53, *P* < 0.01) in the QF. In contrast, the knee flexion peak torque was correlated with RMS (*r* = 0.53, *P* < 0.05) but not with MT and EI in HM. In addition, EI and RMS in QF, and RMS in HM were selected as the major determinants of muscle strength in the stepwise regression analysis. These results suggest that muscle strength is moderately associated with different factors related to the thigh muscles in young individuals.

## Graphical abstract

This study investigated the relative contribution of neuromuscular activation pattern, muscle size, and muscle quality to the maximum strength of quadriceps femoris and hamstring muscles. The results showed a correlation between the knee flexion peak torque and the root mean square as the parameter of neuromuscular active pattern; another correlation between the knee extension peak torque and the echo intensity as the parameter of muscle quality was shown in this study. These results suggest that muscle strength is moderately associated with different factors related to the thigh muscles in young individuals.

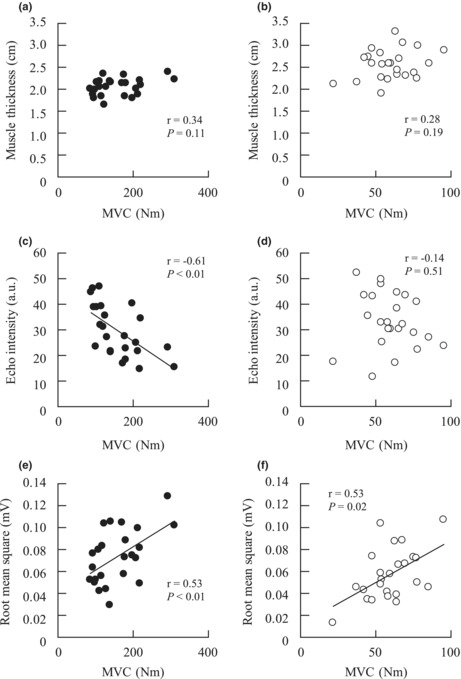

## 1 INTRODUCTION

Muscle strength is a motor output capability and a fundamental parameter for human movement. Previous studies have determined the physiological effects of aging and exercise on skeletal muscles based on muscle strength analyses (Enoka, 1988; Mitchell et al., 2012). Muscle size, such as muscle thickness (MT), cross‐sectional area, and volume, are related to muscle strength (Akagi et al., 2009; Freilich et al., 1995; Maughan et al., 1983). Freilich et al. (1995) showed that MT evaluated by ultrasound imaging was related to maximal muscle strength (*r* = 0.55–0.56, *P* < 0.05). Conversely, the neuromuscular activation pattern (Lawrence & De Luca, 1983), composition of fiber type (Clarkson et al., 1980), and non‐contractile tissue concentration (Goodpaster et al., 2001) are other contributors to muscle strength apart from muscle size. These findings suggest that the effects of these additional factors on muscle strength should be clarified more precisely to understand the individual differences in muscle strength.

Among the contributors to muscle strength, the neuromuscular function is important because voluntary muscle contraction is regulated by the central nervous system. Surface electromyography (EMG) is widely used to measure and quantify neuromuscular function because it can noninvasively detect the signals reflecting motor unit activation properties, which trigger the requisite physiological processes in muscle contraction (Farina et al., 2004; Lawrence & De Luca, 1983). A previous study showed a positive and excellent correlation between motor unit firing rate and maximal muscle strength of knee extensor muscles (Watanabe et al., 2016). Furthermore, the root mean square (RMS) of EMG was selected as the indicator of muscle strength after adding the MT component (Watanabe et al., 2018). From another perspective, excessive concentrations of non‐contractile tissues, such as lipids and/or fibrous tissue, negatively affect muscle strength. Echo intensity (EI) determined by the black‐to‐white color scale from ultrasound images has been used to evaluate non‐contractile tissue concentration (Akima et al., 2016; Reimers et al., 1993). In addition, the relationship between maximal muscle strength and EI has been demonstrated in previous studies (Cadore et al., 2012; Rech et al., 2014). Using training investigation, change in muscle strength was investigated along with changes in MT, RMS, and EI (Ikezoe et al., 2020; Radaelli et al., 2014). These studies showed that the timing of adaptation was inconsistent among MT, RMS, and EI through 8–12 weeks of resistance training. Initially, the adaptation was shown in RMS, after that in MT, and finally in EI. This result showed that the improvement in those neuromuscular factors greatly contributed to the magnitude of muscle strength in the first stage (Moritani & deVries, 1980), suggesting that individual differences in muscle strength in non‐trained individuals are influenced by the RMS, rather than by MT and EI. However, there is a lack of clarity regarding which contributing factor greatly affects the individual differences in muscle strength in non‐athlete young individuals. This knowledge will help elucidate every individual's underlying physiological characteristics of muscle strength. This will be useful for exercise training, preventing age‐related atrophy, and explaining the physical recovery rates.

Previous studies have investigated skeletal muscle strength and related mechanisms using thigh muscles. The quadriceps femoris (QF) and hamstrings (HM) act as agonists and antagonists for respective knee joint movements. In a study by Evangelidis et al. (2016) differences in knee extension strength was estimated as 71% of the QF size and that of the knee flexion was 38% of the HM size. The muscle size was considered to have a different influence on muscle strength, depending on the muscle group. Furthermore, the content of intramuscular adipose tissue (IntraMAT), a non‐contractile tissue closely related to the EI value, was investigated by Akima et al. (2015) in QF and HM. In this study, the content of IntraMAT in HM was fourfold greater than that in QF, showing the possibility that non‐contractile tissue concentrations can preferentially explain individual differences in knee flexion strength rather than muscle size. Hence, the priority of candidates to explain muscle strength would differ depending on the muscle group. However, the mechanisms by which neuromuscular activation parameters, muscle size, and non‐contractile tissue concentration contribute to muscle strength in QF and HM remain unknown.

Therefore, this study aimed to investigate the relationship between maximal muscle strength and neuromuscular activation parameters, muscle size, and non‐contractile tissue concentrations in two thigh muscle groups. We hypothesized that the neuromuscular activation parameter is a major factor related to muscle strength because it has a greater contribution to muscle strength in young individuals. Furthermore, we speculated that the contribution of non‐contractile tissues to muscle strength would be greater in HM because a higher ratio of non‐contractile tissue was included in HM.

## 2 MATERIALS AND METHODS

### 2.1 Participants

Twenty‐four young men and women participated in this study (11 men and 13 women; age 20.3 ± 1.3 years; height 162.4 ± 6.6 cm; bodyweight: 57.1 ± 6.6 kg). Before the experiment, the purpose, procedures, and risks associated with the study were explained to each participant, and written informed consent was obtained from all participants. In addition, all the examination protocols were approved by the ethics committee of Chukyo University (no. 2020–058). Furthermore, the study was conducted following the ethical principles of the Declaration of Helsinki.

### 2.2 Maximal isometric knee extension and flexion strength

Maximal voluntary contraction (MVC) was measured. The maximal voluntary isometric knee extension and flexion strengths of the right leg were measured using a dynamometer mounting force transducer (VINE, Tokyo, Japan). The hip was fixed to the dynamometer using a strap, and the knee joint angle was fixed at 90° (0° means fully extended). MVC was exerted from baseline to maximum in 3–4 s and sustained at maximum for 3 s. Two MVC trials were performed, and the maximum force was selected as the MVC. The force signals were sampled at a frequency of 1000 Hz using an analog‐to‐digital converter (PowerLab; ADInstruments, Melbourne, Australia) and the data were stored on a computer (MacBook Pro; Apple Inc., CA, USA). Maximal torque was calculated as the exerted force (N) × lever arm length of the dynamometer (m).

### 2.3 Electromyography measurement

Surface EMG signals from the rectus femoris (RF), vastus lateralis (VL), long head of the biceps femoris (BF), and semitendinosus (ST) were recorded during the maximal isometric knee extension and flexion tasks using active electrodes. A single differential electrode with a 1‐cm interelectrode distance and an input impedance of 10G Ω was used (FAC‐SEMG1, 4 Assist, Tokyo, Japan). The signal from the EMG system was filtered at 20–450 Hz and sampled at 2000 Hz using an analog‐to‐digital converter (PowerLab; ADInstruments, Melbourne, Australia) and stored on a personal computer (Mac Book Pro; Apple Inc., CA, USA) using the Chart 5.5 software (ADInstruments). Before attaching electrodes, the skin was shaved, abraded, and cleaned with alcohol. The locations of the electrodes for individual muscles were determined to correspond to the locations recommended by the Surface ElectroMyoGraphy for the Non‐Invasive Assessment of Muscles. The electrodes were placed in the middle of the right thigh, corresponding to the midpoint between the greater trochanter and lateral condyle. The electrodes for the four muscles (RF, VL, BF, and ST) were placed approximately parallel to the muscle fibers for each muscle. The RMS was calculated for a 1‐s period during the sustained MVC and a 1‐s period during each steady force phase of the intermittent isometric exercise. The RMS in the RF and VL was averaged as RMS in the QF. In addition, the RMS in the BF and ST was averaged as RMS in the HM.

### 2.4 Ultrasound measurements

MT, subcutaneous fat (SF) thickness, and EI of the mid‐thigh were measured using an ultrasound device following a previously published procedure for ultrasound measurement (Yoshiko et al., 2018). The participants were rested in the supine position while fully extending the knee before the ultrasound measurements to avoid muscle contraction‐induced blood flow and fluid shifts (Berg et al., 1993). After a 10‐min rest, we scanned four regions. The anterior and lateral regions were imaged on the ultrasonography device in the supine position, while the two posterior regions (used for observing BF and ST) were imaged with the participant in the prone position with fully extended knee joints. In addition, we performed the ultrasound measurements in the same location of EMG measurement in the right mid‐thigh, corresponding to the midpoint between the greater trochanter and lateral condyle. A B‐mode ultrasonography device (LOGIQ e premium; GE Healthcare Japan, Tokyo, Japan; 3.8‐cm width) and 4.2‐ to 13.0‐MHz linear array probe (L4‐12t‐RS; GE Healthcare Japan, Tokyo, Japan) were used to obtain images with the following acquisition parameters: frequency, 10 MHz; gain, 35 dB; depth, 3.0–5.0 cm; and focus point, 1 (top of the image). Depth was determined for every participant, and it was generally ≤5.0 cm. A water‐soluble gel was applied to the scanning head of the probe to achieve acoustic coupling. The measurements were performed with utmost care to avoid any deformation of muscle morphology by placing the probe on the skin surface. Three images of each section were stored in the Digital Imaging and Communications in Medicine format and transferred to a personal computer for analysis with the ImageJ software, version 1.46 (National Institutes of Health, Bethesda, MD, USA). MT of the RF, VL, BF, and ST was defined as the distance between the inferior boundary of the surface fascia and upper boundary of the deep fascia. The MT in RF and VL was averaged as MT in QF, and MT in BF and ST was averaged as MT in HM. SF was defined as the distance between the dermis and lower boundary of the surface fascia.

EI was assessed based on the pixel‐by‐pixel 256 gray scale level using the ImageJ software and expressed in arbitrary units (a.u.). In addition, we set the region of interest by tracking the largest possible outline of the muscle while excluding the visible fascia and bone in the RF, VL, BF, and ST. The mean EI within the region of interest in the RF, VL, BF, and ST was calculated for each image. The EI in the RF and VL was averaged as EI in the QF, and EI in the BF and ST was averaged as EI in the HM.

### 2.5 Statistical analysis

All values are reported as means ± standard deviation. First, we performed a Shapiro–Wilk test to confirm the normality of the parametric data. Subsequently, Pearson's product–moment correlation coefficient was used to determine the relationship between peak torque, RMS, MT, and EI using the correlation coefficient (*r*). In addition, stepwise multiple regression analysis was performed with knee extension or flexion peak torque as the dependent variable and RMS, MT, and EI in QF or HM as independent variables. To avoid multicollinearity in the analysis, we checked that the variance inflation factor was lower than the criteria (i.e., 10) in all stepwise regression analyses. Furthermore, the level of significance was set at *P* < 0.05. All statistical analyses were performed using the SPSS Statistics version 22.0 J (IBM Japan, Tokyo, Japan).

## 3 RESULTS

The participants' MT, EI, and RMS in the thigh muscles are shown in Table 1. The thicknesses of the SF were 0.71 ± 0.22 cm in the anterior thigh, 0.57 ± 0.21 cm in the lateral thigh, and 0.94 ± 0.37 cm and 0.77 ± 0.27 cm in the two posterior thigh regions. The knee extension peak torque was 159.8 ± 61.6 Nm, and the knee flexion peak torque was 59.9 ± 16.0 Nm. A significant correlation with knee extension peak torque was found for EI (*r* = −0.61, *P* < 0.01) and RMS (*r* = 0.53, *P* < 0.01) but not for MT (*r* = 0.34, *P* = 0.11) in QF. There was a significant correlation between the knee flexion peak torque and RMS in the HM group (*r* = 0.53, *P* = 0.02).

**TABLE 1:** Muscle thickness, echo intensity, and root mean square in all participants (*n* = 23)

	RF	VL	QF	BF	ST	HM
Muscle thickness (cm)	1.97 ± 0.19	2.15 ± 0.24	2.06±0.19	2.87 ± 0.38	2.25 ± 0.39	2.56 ± 0.34
Echo intensity (a.u.)	29.7 ± 11.1	29.1 ± 10.5	29.4 ± 10.0	29.2 ± 11.5	38.0 ± 12.1	33.6 ± 10.9
Root mean square (mV)	0.08 ± 0.02	0.07 ± 0.03	0.07 ± 0.02	0.05 ± 0.02	0.06 ± 0.03	0.06 ± 0.02

*Note*: Root mean square (RMS) of RF, VL, and QF was calculated during the knee extension task. The RMS of BF, ST, and HM was calculated during the knee flexion task.

Abbreviations: BF, biceps femoris; HM, hamstrings; QF, quadriceps femoris; RF, rectus femoris; ST, semitendinosus; VL, vastus lateralis.

In the stepwise regression analysis for knee extension peak torque, the EI and RMS of the QF were selected from three independent variables (Table 2). The final regression equation was as follows: knee extension peak torque (Nm) = −2.983 × EI of QF (a.u.) + 915.727 × RMS of QF (mV) + 180.585. The standardized coefficients of determination (*R* ^2^) and adjusted *R* ^2^ values were 0.489 and 0.441, respectively. The RMS of the HM was selected as an independent variable for the regression of knee flexion peak torque (Table 3). The final regression equation was as follows: knee flexion peak torque (Nm) = 356.546 × RMS of HM (mV) + 39.509. The standardized coefficients of determination (*R* ^2^) and adjusted *R* ^2^ values were 0.276 and 0.243, respectively.

**TABLE 2:** Stepwise regression analysis as a dependent variable of the knee extension peak torque

	Independent variables	B	Constant	*Β*	*R* ^2^	Adjusted *R* ^2^	*P*
Model 1	EI	−3.715	269.072	−0.605	0.366	0.337	<0.001
Model 2	EI	−2.983	180.585	−0.486	0.489	0.441	<0.001
	RMS	915.727		0.371			

*Note*: Independent variables were muscle thickness in quadriceps femoris (QF), echo intensity (EI) in QF, and root mean square (RMS) in QF.

Abbreviations: B, Non‐standardizing coefficient; *β*, standardized regression coefficient; *R* ^2^, coefficient of determination.

**TABLE 3:** Stepwise regression analysis as a dependent variable of the knee extension peak torque

	Independent variables	B	Constant	*β*	*R* ^2^	Adjusted *R* ^2^	*P*
Model 1	RMS	356.546	39.509	0.525	0.276	0.243	0.008

*Note*: Independent variables were muscle thickness in hamstrings (HM), echo intensity in HM, and root mean square (RMS) in HM.

Abbreviations: B, Non‐standardizing coefficient; *β*, standardized regression coefficient; *R* ^2^, coefficient of determination.

## 4 DISCUSSION

We investigated the relationship between the maximum muscle strength, neuromuscular activation parameters, and size and quality of the QF and HM. We found that (1) the knee extension peak torque was correlated with EI and RMS of QF, and the knee flexion peak torque was correlated with the RMS of HM. (2) EI and RMS of QF and RMS of HM were selected as the primary variables to determine knee extension and flexion muscle strength, respectively, by stepwise regression analysis. These results suggest that muscle strength is determined by different thigh muscle candidates in young non‐athletic individuals.

Many studies have evaluated the effects of aging, disease, and exercise on muscle strength. Furthermore, maintaining higher muscle strength during the young and middle ages is important because muscle strength declines with age, especially after 50 years (Larsson et al., 1979). In addition, muscle size is related to muscle strength (Akagi et al., 2009; Freilich et al., 1995; Maughan et al., 1983).

**FIGURE 1:**
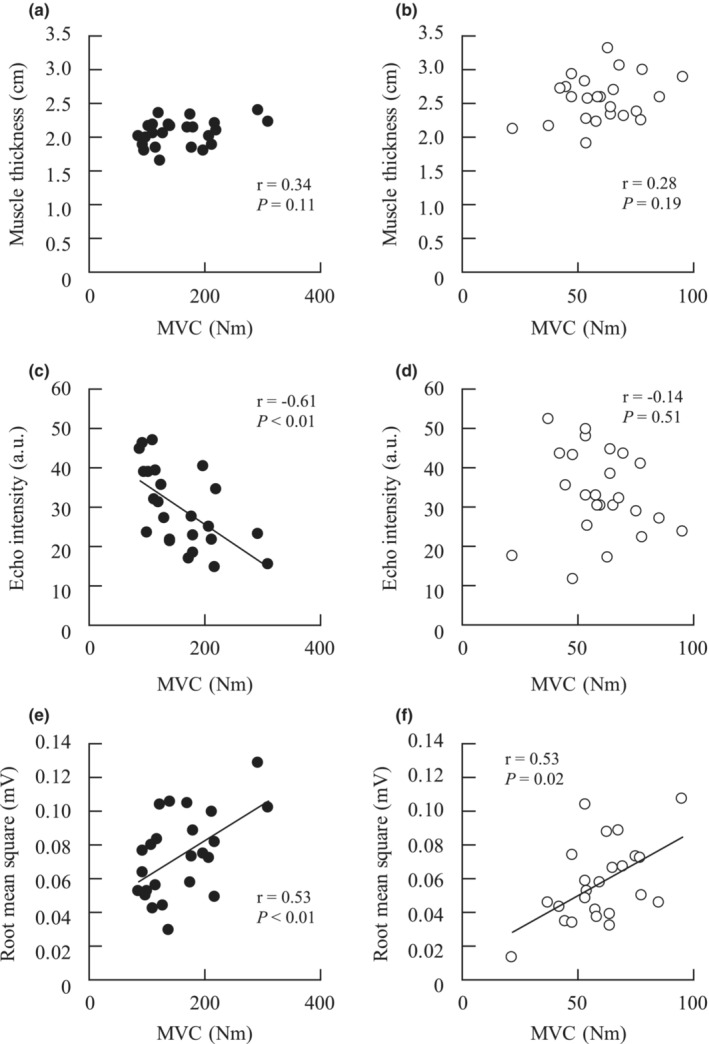
Relationships of the maximum strength during maximal voluntary contraction (MVC) with the muscle thickness (MT), echo intensity (EI), and root mean square (RMS) of the quadriceps (a, c, e) and hamstrings (b, d, f).

We measured the MT from ultrasound images as a parameter of muscle size in this study because this measurement is technically simple, cost‐effective, and non‐invasive. Contrary to our hypothesis, we found no relationship between MT in QF and HM and maximal muscle strength (Figure 1a,b). MT has been used to indicate muscle size, especially in older individuals (Cadore et al., 2012; Radaelli et al., 2014; Yoshiko et al., 2018). Watanabe et al. (2018) showed a relationship between maximal muscle strength and MT in older individuals; however, this relationship was not found in young individuals. To the best of our knowledge, only a few studies have shown that MT is significantly related to maximal muscle strength in young individuals (Freilich et al., 1995; Strasser et al., 2013). Considering these findings, this relationship needs to be clarified in young individuals, and the importance of MT in this population should be reconsidered. From another perspective, there were two technical limitations for assessing MT: (1) although QF and HM consist of four and three muscles (RF, VL, vastus intermedius, and medialis in QF, and BF, ST, and semimembranosus in HM), we could not evaluate the MT of all muscles; (2) MT information was limited compared with other muscle size indicators, such as muscle cross‐sectional area and mass. This is because MT indicates one‐dimensional information about muscle size. In contrast, Miyatani et al. (2004) established a formula that examines muscle mass from MT with high accuracy. Therefore, our technical limitations can be overcome using this formula. Furthermore, we should consider using other assessment tools while analyzing device accessibility.

The EI determined by the black‐to‐white color scale from ultrasound images has been used to evaluate non‐contractile tissue concentrations, and it was significantly related to the IntraMAT content measured by MRI and extramyocellular lipids measured by magnetic resonance spectroscopy (Akima et al., 2016). Excessive lipid accumulation in the muscle is associated with impaired muscle fiber contraction and lower force development (Choi et al., 2016; Rahemi et al., 2015). Therefore, assessing the non‐contractile tissue concentration is more important as a skeletal muscle feature. Studies by Cadore et al. (2012) and Rech et al. (2014) showed a relationship between maximal strength and EI in older individuals, which is less investigated in young individuals. Mota and Stock (2017) found that the magnitude of the relationship between the maximal strength and EI was moderate but not significant (*r* = −0.56, *P* = 0.06) in young men (age, 26 ± 3 years; body mass index, 65.2 ± 8.8 kg/m^2^). They mentioned that this result was due to the small sample size (the sample size was 12). To partly support this result, we found a moderate relationship between muscle strength and EI in the QF (r = −0.61, *P* < 0.01, Figure 1c), but not in the HM (*r* = −0.14, *P* = 0.51, Figure 1d). This result indicated that EI is an important candidate for maximal knee extension strength, even in young individuals. Furthermore, we should discuss why this relationship was not observed in HM. Few studies have investigated EI in HM. One study showed that EI in the BF was related to RF (*r* = 0.49, *P* < 0.05), implying that the pattern of individual differences in EI was similar in the QF and HM (Yoshiko et al., 2018). Importantly, it is well known that EI is affected by surface layer thickness, for example, SF thickness. To confirm the effect of SF, we additionally examined the difference in SF among the anterior, lateral, and two posterior thigh regions using a one‐way analysis of variance (unpublished data). The SF in the posterior thigh regions (over the BF) was significantly thicker than that in the anterior and lateral thighs (*P* < 0.05). Therefore, the influence of SF thickness on EI would be greater in HM than in QF because of higher subcutaneous fat thickness (Neto Müller et al., 2021; Ryan et al., 2016; Young et al., 2015). Young et al. (2015) showed that the EI in BF explained 20% of IntraMAT measured by MRI before correction by subcutaneous fat thickness; however, the corrected EI explained 64% of IntraMAT. Therefore, more attention should be paid to deal with EI in HM as a parameter of non‐contractile tissue concentration.

We observed that RMS and maximal muscle strength showed a significant relationship in knee extensors and flexors (Figure 1e,f). In addition, RMS was selected as an independent variable to explain the maximal muscle strength (Tables 2 and 3). This result supports our hypothesis and previous finding (Watanabe et al., 2018). The EMG amplitude variables, that is, RMS, mainly reflect the number of recruited motor units and/or their firing rate during voluntary contraction. It is reasonable to conclude that neuromuscular function contributes to muscle strength because voluntary muscle contraction is regulated by the central nervous system. Previous studies have demonstrated that after exercise training, changes in the muscle strength accompanied changes in MT, RMS, and EI (Ikezoe et al., 2020; Radaelli et al., 2014). These studies showed that with an increase in strength, the RMS signal first improved, followed by improvements in the MT and EI. This result supports the hypothesis that neural factors greatly contribute to the magnitude of muscle strength in young individuals (Moritani & deVries, 1980). Our results further suggest that the neuromuscular activation parameter would be a higher contributing factor to muscle strength than muscle size and non‐contractile tissue concentration. However, the balance of this contribution may break in athletes. Moritani and deVries (1980) showed that muscle size was a greater contributor to muscle strength than neural factors in accordance with training; thus, whether this contribution order would change by a training intervention would be a good starting point for further research.

Using stepwise multiple regression analysis, EI and RMS were selected as independent variables to explain maximal knee extension strength (Table 2). The standardized regression coefficient of the regression analysis implied the contribution of each variable to the regression. EI was approximately 10% greater in explaining the variation compared with RMS (EI, −0.486; RMS, 0.371) for knee extension strength. This result implied that non‐contractile tissue concentration is one of the variables for individual differences in muscle strength compared with neuromuscular activation patterns in young individuals. EI has been used to evaluate non‐contractile tissue concentrations, especially muscle lipids, such as IntraMAT (Akima et al., 2016). Recent studies have found that excessive muscle lipid accumulation impairs muscle fiber contraction (Choi et al., 2016; Rahemi et al., 2015). Furthermore, Eshima et al. (2020) found a relationship between the droplet size of muscle lipids and the Ca^2+^ release capacity. These findings may indicate an underlying mechanism for the negative relationship between non‐contractile tissue and muscle force production. One previous study showed a negative relationship between IntraMAT and central activation levels in older individuals (Yoshida et al., 2012). The result implies that candidates for muscle strength would interfere. However, it is still unknown whether this could be applied to explain the individual strength differences in our young participants because the previous findings were observed in participants with specific physical characteristics, such as older individuals and obesity. Our study could not identify these underlying mechanisms; therefore, they should be clarified in further studies.

Our study has some limitations. First, we investigated three parameters—MT, EI, and RMS— as factors contributing to force production leading to torque in the knee joint. Muscle torque has been used as a parameter to determine muscle function, and it is determined by biomechanical, morphological, and quantitative properties in addition to the parameters of MT, EI, and RMS. These properties include different types of muscle fiber components, the content of intramuscular connective tissues, capillary density, and others (Frontera & Ochala, 2015). In this study, after a stepwise regression analysis, 24% to 44% of peak torque was explained using EI and/or RMS (Tables 2 and 3). In other words, the remaining 56% to 76% would be explained by factors affecting muscle strength not included in our analysis. Unfortunately, investigating all factors related to force production is beyond the scope of this study. In future research, we will elucidate contributions to muscle strength using multiple factors to show the mechanism of muscle force production. Second, we needed to consider confounding factors when using correlation and regression analysis. For instance, maximal peak torque and muscle mass depend on the body mass index (BMI), implying peak torque and muscle mass would be higher in participants with higher BMI. Therefore, BMI would become a confounding factor in the relationship between these factors. In this study, we confirmed that the result was not different when BMI was considered as a confounding factor or not using partial correlation (unpublished data). Therefore, we should consider confounding factors more carefully when increasing factors related to muscle force production.

## 5 CONCLUSION

Maximal knee extension and flexion strength were related to RMS in QF and HM. These results suggest that neuromuscular function is important for determining muscle strength. Furthermore, we revealed that the EI and RMS of the QF and RMS of the HM were selected as variables to explain muscle strength by stepwise regression analysis. This result implies that muscle strength is determined by different factors between the thigh muscles in young individuals. In future research, it should be clarified whether this contribution order would change by training intervention and what type, intensity, and frequency of training would better improve neuromuscular function and non‐contractile content, eventually leading to the improvement of muscle strength in young individuals.

## FUNDING INFORMATION

This study was supported by the Nakatomi Foundation of A.Y.

## CONFLICT OF INTEREST

The authors declare that they have no conflicts of interest.
